# Primary laryngeal tuberculosis mimicking laryngeal carcinoma: CT scan features

**DOI:** 10.4103/0971-3026.59745

**Published:** 2010-02

**Authors:** N Ech-Cherif El Kettani, MR El Hassani, N Chakir, M Jiddane

**Affiliations:** Department of Neuroradiology, Hôpital des Spécialités, CHU Ibn Sina, Rabat, Morocco

**Keywords:** Carcinoma, larynx, tuberculosis

## Abstract

Laryngeal tuberculosis is a rare disease. It is almost always associated with pulmonary tuberculosis. It occurs generally in adults without BCG vaccination or in cases of the acquired immune deficiency syndrome. On laryngoscopy and imaging, it often simulates laryngeal carcinoma, and confirmation is always histological. We report the case of a 36-year-old man who presented to our hospital with dysphonia and dysphagia. Laryngoscopy revealed a lesion of the left vocal cord and the ventricular strip. CT scan found focal, regular thickening of the left vocal cord, associated with irregular thickening of the posterior laryngeal wall. A biopsy confirmed the diagnosis of tuberculosis.

## Introduction

Primary laryngeal tuberculosis is very uncommon.[1] It often simulates malignancy on imaging and laryngoscopy, and the final diagnosis is invariably based on biopsy.[1,2]

## Case Report

**Figure 1:**
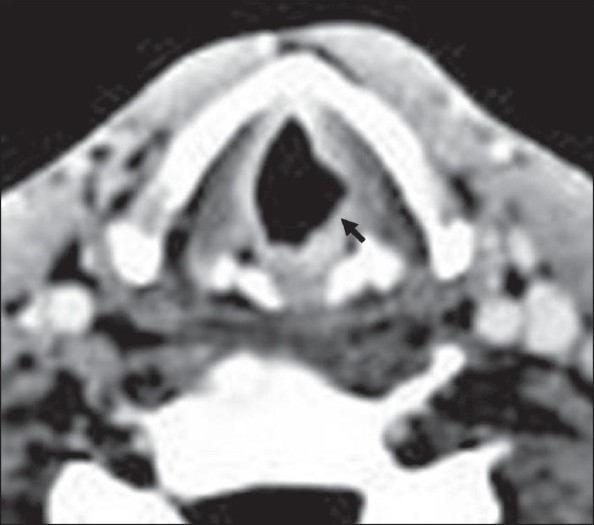
Axial contrast-enhanced CT scan of the larynx shows regular thickening of the left vocal cord (arrow)

**Figure 2:**
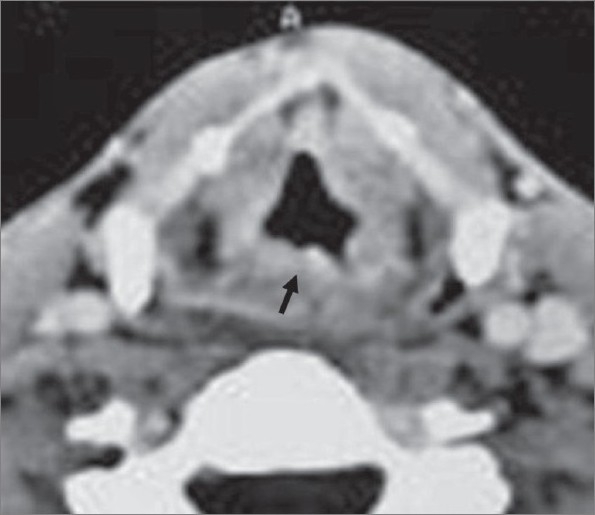
Axial contrast-enhanced CT scan of the larynx shows irregular thickening of the posterior laryngeal wall (arrow)

A 36-year-old-man without a medical history of pulmonary tuberculosis or BCG vaccination was admitted for dysphonia and dysphagia for 6 months. Laryngoscopic examination revealed thickening of the left vocal cord and the ventricular strip, with edema. Laryngeal CT scan [Figures 1 and 2] revealed a localized, regular thickening of the left vocal cord, associated with irregular thickening of the posterior laryngeal wall. The anterior commissure and sub-glottic region were normal. There were neither any cartilaginous abnormalities nor any cervical adenopathy. A biopsy was performed, which revealed caseo-follicular tuberculosis laryngitis. The chest radiograph and abdominal USG were normal. On further interrogation and investigations, it was found that the patient was negative for the human immunodeficiency virus and his sputum was negative for acid-fast bacilli.

## Discussion

Laryngeal tuberculosis is due to *Mycobacterium tuberculosis*. It represents the most frequent laryngeal granulomatous disease, but a primary laryngeal location is exceptional. Indeed, this disease is almost always associated with pulmonary tuberculosis.[1–3]

Laryngeal tuberculosis tends to mimic laryngeal cancer, and its laryngoscopic appearance often simulates malignancy. It usually occurs due to reactivation of a laryngeal focus that may have appeared during hematogenous dissemination of a tuberculosis primary infection. It occurs mostly in adults aged between 40 and 50 years, who complain of progressive and insidious hoarseness and odynophagia. Some factors that have been found to occur in these patients include the absence of BCG vaccination and the presence of malnutrition, promiscuity, aquired immunodeficiency syndrome, immunosuppression and tobacco use.[1,4]

On laryngoscopy, tuberculosis may either present as a mass or as an ulcer. The location of the lesion is extremely variable. The vocal cords represent the most frequent site, followed by the ventricular strip, epiglottis, sub-glottic region and posterior commissure. A posterior location is known as well. The lesion is bilateral in 75% of the cases.[4,5]

The radiological findings of laryngeal tuberculosis depend on the stage and lesion extension, and these correlate directly with the histological findings. In the infiltrative stage, there is focal thickening. In the ulcerative stage, the ulceration is not deep and rarely reaches the paraglottic spaces and the cartilage. Perichondritis is sometimes noted (epiglottis, arytenoids), but calcifications are not common and the para-laryngeal fat spaces are usually spared. The last stage is characterized by sclerosis. Various radiological findings that have been described include edema alone, an ulcero-infiltrative mass, infiltrative and pseudo-tumoral appearance (66%); sub-glottic laryngitis (isolated swelling of the ary-epiglottic fold or even massive cartilaginous ulceration and, sometimes, chondritis or perichondritis); diffuse form; and tuberculoma (enormous ventricular vegetation with a large base that elevates the ventricular strip).[5–6]

Laryngeal carcinoma is the main differential diagnosis. It is usually not possible to distinguish between these two conditions on imaging.

Finally, treatment is based on anti-tuberculosis chemotherapy, but surgery is indicated in the presence of laryngeal stenosis.[5]
